# 1256. Antiretroviral Switching in Colombia: A Retrospective Cohort Study.

**DOI:** 10.1093/ofid/ofac492.1087

**Published:** 2022-12-15

**Authors:** Shirley Vanessa Correa Forero, Sandra Valderrama, Samuel Martínez-Vernaza

**Affiliations:** Hospital Universitario San Ignacio, BOGOTA, D.C., Distrito Capital de Bogota, Colombia; Hospital Universitario San Ignacio, BOGOTA, D.C., Distrito Capital de Bogota, Colombia; Hospital Universitario San Ignacio,Grupo de Investigación en Enfermedades Infecciosas HUSI-PUJ, Bogota, Distrito Capital de Bogota, Colombia

## Abstract

**Background:**

Antiretroviral therapy (ART) for the human immunodeficiency virus (HIV) has improved life expectancy in people living with HIV/AIDS (PLWHA). Between 2.4% and 50% of PLWHA who initiate ART require a regimen switch within the first year. This variable range depends on local treatment guidelines, available ART, and the prevalence of resistance. We aimed to analyze the causes of ART Switch, time to ART switch and its associated factors in a Colombian Cohort.

1. a. Kaplan Meier Curves and Log-Rank test for time to antiretroviral therapy switch by sex in a cohort of people living with HIV in Colombia 2017-2019. Log-rank test, p: 0.0091. b. Kaplan Meier Curves and Log-Rank test for time to antiretroviral therapy switch by age in a cohort of people living with HIV in Colombia 2017-2019. Log rank test, p: <0.0001. c. Kaplan Meier Curves and Log-Rank test for time to antiretroviral therapy switch by CDC Stage in a cohort of people living with HIV in Colombia 2017-2019. Log rank test, p: 0.006. d. Kaplan Meier Curves and Log-Rank test for time to antiretroviral therapy switch by CD4 cell count in a cohort of people living with HIV in Colombia 2017-2019. Log rank test, p: 0.096

**Figure:**
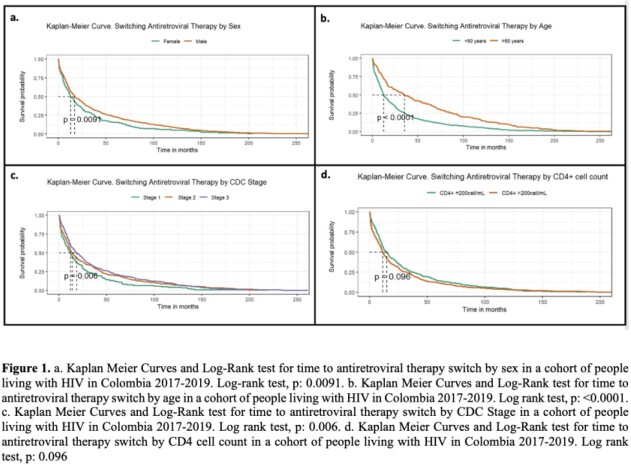

**Figure:**
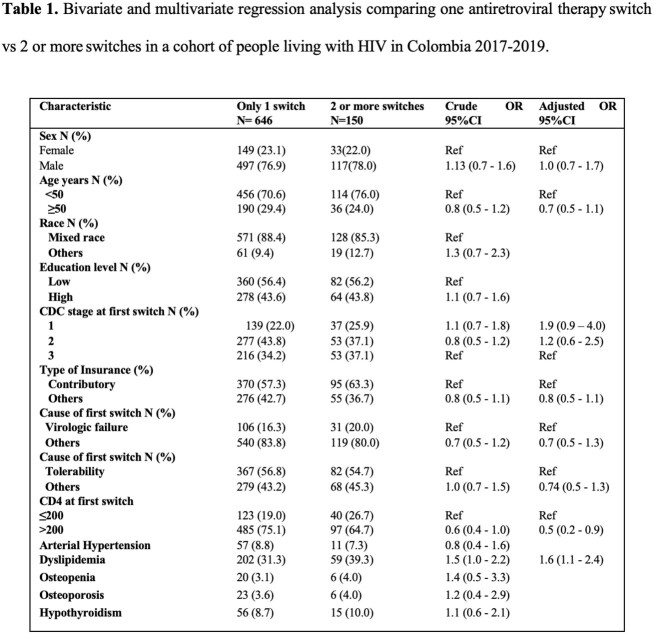

**Methods:**

We conducted a retrospective cohort study in 20 different HIV clinics including patients ≥18 years with confirmed HIV who underwent ART switching from 2017 to 2019 and with at least 6 months of follow up in their HIV Clinic. A time to event analysis and an exploratory Cox model were performed. Also, we conducted a logistic regression for comparing people with only one switch versus those who had 2 or more switches.

**Results:**

796 patients switched ART during the study period. Most of the patients were man (77.1%) and younger than 50 years (71.6%). The main cause of ART switch was tolerability (56.4%) followed by virologic failure (17.2%). Tolerability had a median time to switch of 12.2 months being the shortest, whereas the longest median time to switch was due to simplification (42.4 months). The Kaplan Meier curve and log-rank test for time to antiretroviral therapy switch by age, sex, CD4 count, and CDC stage are shown in **Figure 1.** In the Cox model we found that people with 50 years or older (HR= 0.6; 95% CI (0.5-0.7) and CDC stage 3 (HR= 0.8; 95% CI (0.6-0.9) had less hazard for switching ART over time. When comparing patients with only one ART switch versus those who had two or more ART switches, we found that having dyslipidemia increased the odds of switching ART more than one time (aOR=1.6; 95%CI (1.1-2.4). As opposed to having CD4 counts greater than 200 cell/mL which decreases the odds of having more than one ART switch. (aOR= 0.5; 95%CI (0.2-0.9) **Table 1**.

**Conclusion:**

In this Colombian cohort, tolerability was the main cause of ART switch and the time to change the first ART is shorter than reports in other countries. Dyslipidemia and having CD4 counts smaller than 200 cells/mL increased the odds for requiring two or more changes. In Colombia is crucial to apply current recommendations of ART initiation in order to choose regimens with a better tolerability profile.

**Disclosures:**

**Sandra Valderrama, Infectologa**, MSD: Advisor/Consultant.

